# The influence of constellation virtual community atmosphere on blogger trust and constellation infatuation behavior

**DOI:** 10.1186/s40359-025-02468-8

**Published:** 2025-02-18

**Authors:** Qingxia Li, Shuang Li, Qiang Li, Yingji Li

**Affiliations:** 1 School of Labor Economics, Capital University of Economics and Business, Beijing, 100070 China; 2 Yibin City Commercial Bank, Yibin, 645154 China; 3 School of Economics and Management, Shanghai Technical Institute of Electronics & Information, Shanghai, 201411 China; 4 School of Humanities and Management, Yunnan University of Chinese Medicine, Kunming, 650500 China

**Keywords:** Constellation, Virtual community atmosphere, Blogger trust, Constellation infatuation

## Abstract

With the rapid development of social media, astrology-related virtual communities have gained significant popularity in China. However, the mechanisms behind the formation of astrological addiction behaviors within virtual communities remain largely unexplored. This study, based on the Stimulus-Organism-Response (S-O-R) theory, investigates how community environmental characteristics and user traits influence astrological addiction through the mediation of blogger trust. The results show that both community atmosphere (CA) and community expertise (PK) significantly influence constellation infatuation (CI) through the mediation of blogger trust (BT). Specifically, community atmosphere and community expertise positively affect constellation infatuation through blogger trust, supporting the proposed hypotheses. However, online participation (NI) does not have a significant indirect effect on constellation infatuation via blogger trust. The findings suggest that blogger trust plays a critical role in shaping astrological addiction behaviors within virtual communities. This study offers theoretical insights into community management and contributes to understanding the dynamics of addictive behaviors in online environments.

## Introduction

Astrology is a form of divination introduced from the West, sharing similarities with Chinese traditional Zi Wei Dou Shu. As a cultural system based on correlating birth dates with celestial movements, astrology fulfills people’s spiritual needs for self-knowledge and destiny interpretation through predictions about personality traits, emotional tendencies, and work-social relationships [1]. Research shows that astrology’s widespread popularity stems from its unique mystical appeal and practical value [2], becoming a significant topic for entertainment and daily discussion [3].

The spread of astrology in China has undergone significant evolution. During the transition from traditional media to digital platforms, the rise of various astrology-themed websites, apps, and social media platforms has enabled users to access astrological content more conveniently and deeply [4–5]. Social media, as an emerging communication channel, has not only redefined how users access astrological information but also created unique interaction patterns [6]. Through diverse content forms such as images and short videos, astrology has gained broader dissemination on digital platforms [7]. Data shows that as of August 2023, the number of users with “astrology” in their profiles on Weibo alone reached 770,000, a significant increase from 400,000 in 2019 [8]. Leading bloggers have successfully accumulated massive followings through unique content marketing strategies and contextualized expression, utilizing social platform interaction features [9].

Users demonstrate strong trust in bloggers on social media platforms and engage in frequent interactions through likes, comments, and shares, forming a unique “astrological addiction” phenomenon. Specifically, astrological virtual communities exhibit three notable characteristics: First, communities develop unique interactive atmospheres where users establish close connections through knowledge sharing, strengthening community cohesion [10]; Second, users show high trust in bloggers and maintain continuous engagement in community activities [2]; Third, users’ community trust directly influences their consumption and behavioral decisions, including shopping choices and interpersonal interactions [3]. Academia has conducted multidimensional research on the underlying mechanisms of this “astrological addiction” phenomenon. Studies reveal that astrological culture consumption has evolved into a form of social practice, transcending simple information-seeking needs [1]. Users in virtual communities seek not only fortune guidance but also emotional support and identity recognition through astrological topics [11]. Research on heavy astrology users shows that astrology bloggers have acquired social roles similar to life coaches [12], with their advice significantly influencing users’ important life choices in career planning and relationship decisions [13].

However, existing research has limitations in explaining this phenomenon. While numerous studies have explored the influence of virtual community atmosphere on user behavior, there is limited research on the mechanism in the specific cultural context of astrology. Additionally, existing literature on blogger trust formation mechanisms is fragmented, lacking a systematic theoretical framework. Furthermore, there is a lack of holistic research on the complex “atmosphere-trust-addiction” chain, particularly in explaining users’ transition from rational cognition to emotional dependence. Based on these analyses, this study focuses on three core questions: (1) How does the unique atmosphere of astrological virtual communities influence the establishment of blogger trust? (2) What mediating role does blogger trust play between community atmosphere and user addiction behavior? (3) How do user characteristics (such as expertise level and online engagement) moderate this mechanism? Through constructing an integrated model, this study deeply explores the formation mechanism of user addiction behavior in astrological virtual communities, aiming to provide theoretical guidance and practical implications for virtual community operations and social media marketing.

## Literature review

### Theoretical foundation

The Stimulus-Organism-Response (SOR) theory was first proposed by Mehrabian & Russell [14] to explain how environmental factors influence behavioral responses through individual internal states. The theory comprises three core elements: Stimulus (S) refers to external factors that trigger individual responses, Organism (O) represents internal psychological processes, and Response (R) denotes the ultimate behavioral manifestations. Over nearly 50 years of development, SOR theory has been widely applied to explain consumer behavior and user experience, particularly in virtual community contexts [15]. Recent research has further validated the mechanism by which environmental characteristics influence user behavior through emotional states [16]. Studies have identified platform interactivity, personalized recommendations, and trust as key stimulus factors [15].

Scholars have expanded the application of SOR theory in social media research. Studies show that social interaction and knowledge sharing in virtual communities significantly influence users’ community perceptions [17–18]. High-quality content and opinion leaders’ expertise promote usage intention by enhancing user trust [19]. Community interactivity and trust have been proven crucial factors in promoting user engagement [20–21]. Particularly in user engagement and brand relationships, research has found that community interaction indirectly influences trust building by enhancing users’ sense of virtual community and social capital [22]. Users’ emotional and normative commitment significantly enhance knowledge sharing willingness in virtual communities [23], while community interactivity, sense of participation, and trust are key drivers of user brand engagement [24].

In the context of astrological communities, this study constructs a complete theoretical analysis framework by positioning community atmosphere, online engagement, and expertise as stimulus factors (S), blogger trust as the organism state (O), and astrological addiction as the behavioral response (R). This framework helps systematically understand how external environmental characteristics and user participation lead to the formation of addictive behavior through influencing trust states. Combining existing research findings, positive community atmosphere, deep user engagement, and professional content quality can all serve as important stimulus factors, promoting the formation of addictive behavior by enhancing users’ trust in bloggers.

### Constellation atmosphere

Constellation atmosphere is defined as a relatively stable community environmental characteristic that reflects the supportive and interactive atmosphere created by community managers through specific management rules. Virtual community research has found that community atmosphere is a key factor affecting user participation [25]. Based on psychological climate theory [26], constellation atmosphere includes three key dimensions: community support, community cohesion, and community contribution recognition. Community support manifests as both emotional and informational support [27]; community cohesion reflects common interests and mutual cooperation among members [28]; community contribution recognition emphasizes the recognition of members’ efforts [29]. In the context of rapid social media development, a positive community atmosphere is crucial for maintaining user relationships. Research shows that a supportive atmosphere can significantly enhance user trust levels and participation [25]. A good community atmosphere not only promotes interaction among members but also strengthens users’ trust in content [30]. As a special knowledge-based community, the atmosphere in astrological communities is closely related to users’ emotional attachment and sense of identification [27], playing a positive role in promoting trust building and knowledge sharing among users [31].

### Network involvement

Network involvement refers to users’ continuous participation tendency in the network environment, reflected in dimensions such as activity level, participation depth, and interaction frequency [32]. In the digital age, network involvement has become increasingly important as a key variable for understanding user behavior. Big data analysis research shows that network involvement plays a crucial role in user behavior prediction models, effectively explaining behavioral differences in social networks [33]. Research finds that high-involvement users exhibit three significant characteristics: sustained attention investment, deep cognitive processing, and frequent social interaction [34]. Through deep participation and social interaction, these users not only demonstrate higher levels of social trust and participation willingness [25] but are also more likely to develop lasting emotional connections [30]. Particularly in social media environments, longitudinal studies have confirmed significant causal relationships between network involvement and users’ emotional attachment and behavioral dependence [35].

### Professional knowledge

Professional knowledge in astrological communities refers to the professional level, practicality, and credibility of shared information, which significantly impacts user engagement [36]. Professional knowledge primarily comprises three core elements: theoretical knowledge base, practical application ability, and problem-solving skills, which influence user behavior through both cognitive and emotional pathways [37]. High-quality professional content requires both solid theoretical foundation and practical value, crucial for promoting user participation and community interaction [38]. In knowledge-based communities, the display and dissemination of professional knowledge can directly influence users’ cognitive processes and behavioral choices [39]. Research indicates that systematic professional knowledge can significantly enhance user learning engagement and promote community interaction [40].

### Blogger trust

Blogger trust refers to users’ comprehensive evaluation of both cognitive and emotional trust in astrology bloggers. Research shows that this trust is established through continuous interaction and value identification, displaying relatively stable characteristics [41]. Blogger trust helps users form stable cognitive evaluations and emotional connections, promoting user participation through trust mechanisms [42]. In astrological communities, bloggers as opinion leaders have significant influence on user behavior. Studies find that effective blogger trust can enhance users’ psychological identification and improve community participation quality. Cognitive trust helps users accept bloggers’ professional views; emotional trust promotes emotional connections between users and bloggers; behavioral trust manifests as users’ willingness to follow and adopt bloggers’ suggestions [43]. According to social identity theory, blogger trust can be divided into two dimensions: cognitive trust, including evaluation of bloggers’ professional capabilities and content credibility; and emotional trust, referring to users’ emotional dependence and value identification with bloggers [44].

### Constellation infatuation

Constellation infatuation is defined as a relatively stable, continuous state of cognitive investment and emotional attachment [45]. Research shows that this infatuation tendency is independent of general network usage behavior, presenting unique psychological characteristics and behavioral patterns [46]. Constellation infatuation primarily manifests through three dimensions: continuous attention investment, deep emotional attachment, and frequent interactive behavior [47]. In astrological communities, users with high infatuation tendencies show significant behavioral characteristics. Research indicates that these users often actively engage in information searching, content interpretation, and community participation [48]. Compared to regular users, highly infatuated users demonstrate cognitive investment through continuous knowledge acquisition and interpretation, emotional attachment through deep community relationships, and maintain high-frequency interactions in astrological communities [49].

### Relationships between relevant variables

#### Relationship between constellation atmosphere and blogger trust

Constellation Atmosphere originates from the extension of organizational climate theory in virtual communities [50]. Organizational climate represents environmental characteristics collectively perceived by members, including dimensions of norms, support, and recognition [51]. In virtual community research, atmosphere is considered a crucial factor influencing trust development [52]. A positive organizational climate promotes trust formation among members, primarily through dimensions of Community Support, Community Cohesion, and Community Recognition [53]. The pathways through which virtual community atmosphere influences trust are mainly reflected in emotional connection, cognitive identification, and behavioral interaction [46]. In the context of constellation communities, a positive community atmosphere helps eliminate users’ psychological barriers, enhance emotional exchange, and thereby promote trust building in bloggers. Therefore, this study proposes:

H1: Constellation Atmosphere has a significant positive effect on Blogger Trust.

#### Relationship between network involvement and blogger trust

Network Involvement, based on user participation theory, reflects the depth and breadth of users’ online activities [54]. Network Involvement is defined as the degree and scope of user participation in online activities, which can be operationalized from behavioral dimensions into three aspects: activity level, participation depth, and interaction frequency [55]. Based on social learning theory, continuous social interaction is an important foundation for building trust [56]. In virtual communities, high Network Involvement implies more participation opportunities and interactive experiences, which help deepen understanding and cognition of others [57]. From a mechanical perspective, deep participation promotes trust through enhanced cognitive understanding, frequent interaction cultivates trust through deepened emotional connections, and continuous activity consolidates trust through behavioral reinforcement [58]. Network involvement behaviors in virtual communities have a significant promoting effect on trust building [59]. Therefore, this study proposes:

H2: Network Involvement has a significant positive effect on Blogger Trust.

#### Relationship between network involvement and constellation infatuation

Network Involvement, based on user participation theory, reflects the psychological dependence state caused by deep network participation [54]. From the perspective of constituent dimensions, Network Involvement includes three core characteristics: continuous attention, emotional attachment, and behavioral fixation, which together shape users’ network participation patterns and psychological states [60]. Empirical research shows that there is a significant positive correlation between network usage intensity and dependence level - the higher the frequency and depth of use, the more obvious the user’s dependence tendency [61]. From the perspective of action mechanisms, continuous participation can form stable behavioral inertia, deep interaction will stimulate sustained emotional needs, and frequent contact can strengthen existing cognitive patterns. These three pathways jointly promote the formation and fixation of network involvement behaviors [62]. In virtual communities, users with high Network Involvement often show strong dependence tendencies on community activities and interactions, including both the need for information acquisition and expectations for social connections [63]. Therefore, this study proposes:

H3: Network Involvement has a significant positive effect on Constellation Infatuation.

#### Relationship between professional knowledge and blogger trust

Professional authority theory emphasizes the core role of content professionalism in trust building, suggesting that professional content presentation can effectively promote users’ trust formation in content producers [54]. In virtual community contexts, professional content promotes trust building through enhancing cognitive authority and practical value, reflecting users’ rational cognition and value assessment of professionalism [64]. The mechanism mainly works through three pathways: reducing users’ uncertainty perception in decision-making processes, enhancing positive expectations for future interactions, and improving content producers’ overall credibility in users’ minds [65]. Extensive empirical research further indicates that Professional Knowledge influences blogger trust establishment and consolidation through three key dimensions: systematic theoretical framework, practical content interpretation, and accuracy of professional predictions [66]. Therefore, this study proposes:

H4: Professional Knowledge has a significant positive effect on Blogger Trust.

#### Relationship between professional knowledge and constellation infatuation

Cognitive dependence theory indicates that professional content leads to sustained dependence, built on deep recognition of professional knowledge value and authority. Specifically, when users are exposed to high-quality professional content over time, they gradually develop cognitive reliance and emotional attachment to content providers [54]. Professional content continuously reinforces users’ cognitive dependence and emotional attachment through its unique authority and scarcity characteristics, eventually forming stable dependent relationships. This dependence is reflected in users’ continuous attention, repeated consumption, and active dissemination of content [67]. In virtual community research, there is a significant correlation between professional interpretation ability and user infatuation tendency - the deeper users’ dependence on professional knowledge, the more obvious their infatuation tendency becomes. This phenomenon is particularly prominent in knowledge-intensive virtual communities, where professional knowledge not only satisfies users’ cognitive needs but also cultivates deep emotional connections through continuous interaction processes [54]. Therefore, this study proposes:

H5: Professional Knowledge has a significant positive effect on Constellation Infatuation.

#### Relationship between Blogger trust and constellation infatuation

The integration of social identity theory and trust-dependence theory provides a solid theoretical foundation for understanding the relationship between trust and infatuation. When users establish deep trust in content producers, they gradually lower psychological barriers, strengthen emotional connections, and ultimately form stable infatuation tendencies. This process is mainly achieved through three pathways: cognitive identification, emotional connection, and behavioral following [68]. In virtual community contexts, trust promotes infatuation formation and deepening by meeting users’ needs for social identity and emotional attachment, driving users from rational evaluation to emotional investment [42]. In virtual community research, Blogger Trust has been proven to significantly influence users’ dependence tendencies through mechanisms such as opinion identification, content dependence, and interactive stickiness [69]. Therefore, this study proposes:

H6: Blogger Trust has a significant positive effect on Constellation Infatuation.

#### Mediating role of Blogger trust: constellation atmosphere and constellation infatuation

Social exchange theory emphasizes trust’s key mediating role in how atmosphere influences infatuation. Positive community atmosphere helps establish deep trust in bloggers by reducing users’ psychological distance and improving interaction quality. This trust then transforms into sustained emotional investment and behavioral dependence, ultimately forming stable infatuation tendencies [70]. Specifically, positive community atmosphere creates an open, inclusive interactive environment, laying the foundation for trust building. Through frequent high-quality interactions, users’ initial trust gradually transforms into sustained trust, leading to emotional attachment and cognitive identification [68]. In virtual community research, positive atmosphere has been proven to significantly influence user loyalty and participation by enhancing user trust. This mechanism is mainly reflected in dimensions of knowledge sharing, emotional investment, and behavioral following [71]. Therefore, this study proposes:

H7: Blogger Trust mediates the relationship between Constellation Atmosphere and Constellation Infatuation.

#### Mediating role of Blogger trust: professional knowledge and constellation infatuation

Professional authority and dependence formation theory provide a theoretical foundation for understanding trust’s mediating role. In social media environments, professional knowledge needs to establish user trust before it can transform into sustained cognitive dependence and emotional investment. This process is mainly reflected in three aspects: first, content creators’ demonstrated professionalism and authenticity can enhance users’ initial trust; second, this initial trust gradually transforms into stable trust relationships through continuous interaction; finally, deep dependence based on trust is formed [72]. In information dissemination processes, trust and relationship commitment promote users’ transition from rational cognition to emotional investment through pathways of cognitive dependence, emotional connection, and behavioral fixation [73]. Social media research shows that professional knowledge significantly influences users’ dependence and infatuation tendencies by enhancing user trust, with trust playing a key mediating role in transforming professionalism into user stickiness [74]. Therefore, this study proposes:

H8: Blogger Trust mediates the relationship between Professional Knowledge and Constellation Infatuation.

#### Mediating role of Blogger trust: network involvement and constellation infatuation

Dependence formation theory explains trust’s mediating role in the process of social media use transforming into behavioral tendencies. Research shows that users can establish initial trust through social media communication and interaction, which is strengthened through continued use and dependence, ultimately forming stable behavioral patterns [75]. In social media environments, trust and relationship commitment drive users from general use to deep participation through multiple pathways including cognitive dependence and emotional connection. Trust has been proven to play a crucial mediating role between usage intensity and behavioral stability [76]. In human-computer interaction research, users’ interaction depth needs to establish stable trust relationships before it can transform into sustained dependence tendencies, with trust playing an irreplaceable mediating role between high interaction levels and behavioral fixation [77]. Therefore, this study proposes:

H9: Blogger Trust mediates the relationship between Network Involvement and Constellation Infatuation.

**Fig. 1:**
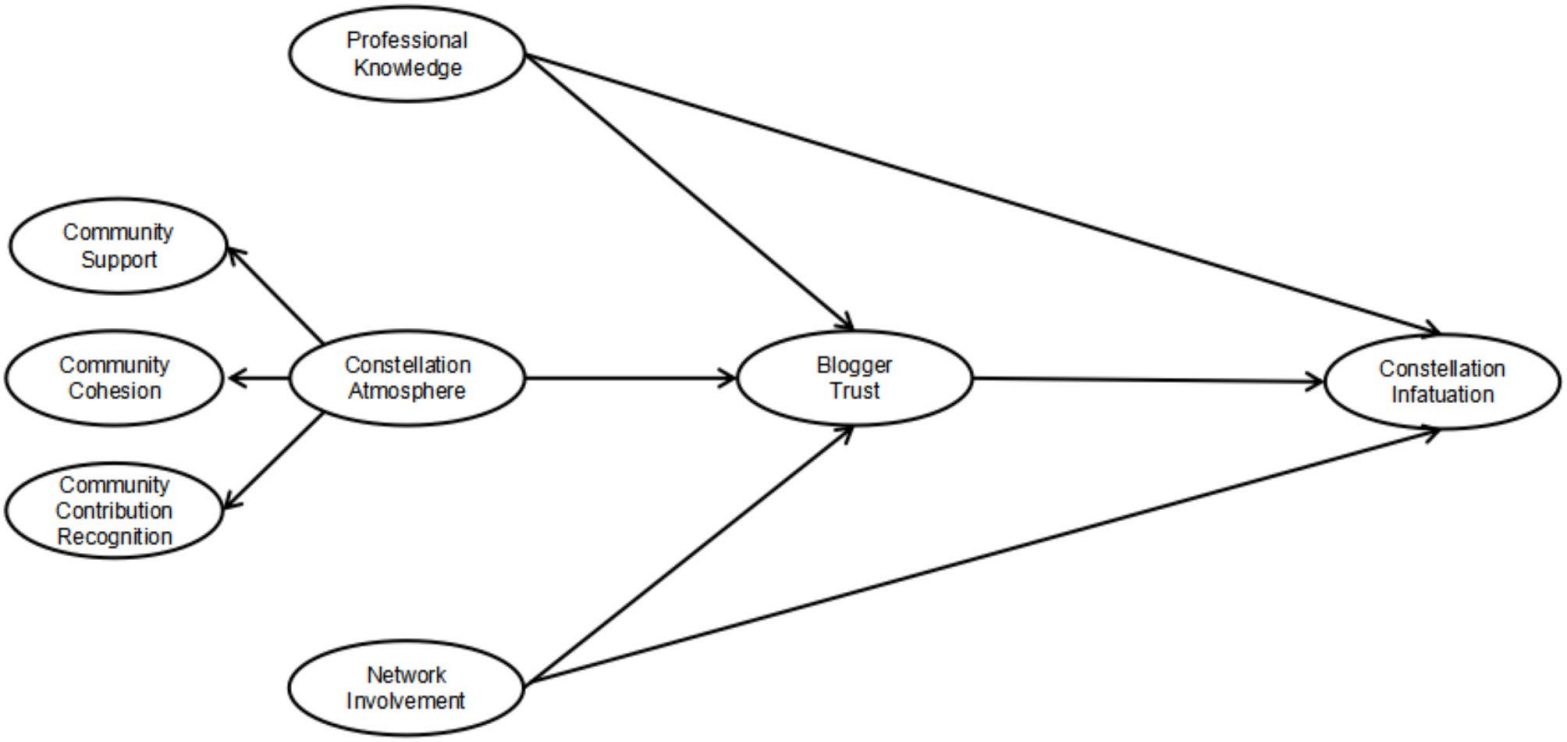
Research model

Based on the above theoretical hypotheses, this study constructs a structural equation model of how constellation virtual community atmosphere affects blogger trust and constellation infatuation behavior (Fig. 1).

## Methodology

This study employs a questionnaire survey method. The following sections detail the sample selection, sample size determination, data collection, research instruments, and data analysis.

### Sample size and data collection

This study focuses on consumers active in constellation communities as research subjects, based on the following considerations: these users have a deeper understanding and experience of constellation culture, enabling them to accurately reflect the characteristics and influences of constellation communities; their frequent interactions with constellation bloggers make them suitable for studying the trust formation mechanisms; as deeply engaged participants, they have thorough perceptions of the community’s professional knowledge and atmosphere, helping to validate the theoretical model proposed in this research. Specifically, research subjects must meet the following criteria: continuous following of constellation bloggers for no less than 3 months, participating in constellation community discussions at least 3 times per week (including reading, liking, commenting, sharing, and other interactive behaviors), and having a basic understanding of constellation knowledge with the ability to comprehend professional terminology and discussion content in the community.

Given that the research purpose specifically targets active users in constellation communities, this study employs purposive sampling method, conducting targeted questionnaire surveys on representative constellation community platforms. The survey was conducted through the “Wenjuanxing” survey website from December 2021 to February 2022, mainly selecting fan groups and super-topic communities of well-known constellation bloggers (such as Tao Baibai, Alex-Uncle) with over 1 million followers on Weibo platform, as well as constellation-themed WeChat and QQ groups with more than 1,000 active members as survey venues. To ensure data quality, strict screening mechanisms were implemented in the questionnaire, including initial screening through questions like “Do you follow constellation bloggers?“, “Are you a member of constellation communities?“, “How many times do you participate in community discussions monthly?“, setting basic constellation knowledge questions to verify respondents’ necessary comprehension abilities, and including reverse questions and logic verification questions to eliminate carelessly completed samples.

A total of 703 questionnaires were distributed in this survey. After strict screening, eliminating samples that did not meet the criteria (including those who did not follow constellation bloggers and those with weekly participation frequency less than 3 times) and abnormal responses, 334 valid questionnaires were obtained, representing an effective response rate of 47.7%. According to the sample size calculation criteria proposed by Wang et al. [78], each measurement variable requires at least 5–10 samples. This study includes 48 measurement items, requiring 240–480 samples by this standard, and the final 334 valid questionnaires fall within this reasonable range. Looking at the sample characteristics (see Table 1), the respondents show the following overall features: predominantly female (85.9%) in terms of gender, mainly concentrated in the 18–25 age group (87.1%), primarily bachelor’s degree or above in education level (86.8%), and mainly full-time students in occupation (77.2%). These demographic characteristics generally align with the current user composition of constellation communities, showing good representativeness. Regarding monthly disposable income, the group with less than 2,000 yuan accounts for the highest proportion (51.5%), which corresponds to the high proportion of students in the sample. Overall, the sample composition reflects the typical user characteristics of current constellation communities, providing a reliable data foundation for testing research hypotheses. The demographic data of the research sample (*N* = 334) is as follows:

### Operational definitions of variables

The constructs (variables) in the research model were defined based on existing related research concepts, adapted to the current research topic, and used as the basis for setting observation indicators and items. The demographic data of the study sample (*N* = 334) are as follows (Table 1):

**Table 1:** Demographics data

Demographic variables	Category	Frequency	Percentage (%)
Gender	Male	47	14.1
	Female	287	85.9
Age	Under 18	12	3.6
	18–25	291	87.1
	26–30	25	7.5
	31–40	6	1.8
Monthly disposable income	Below 2000	172	51.5
	2000–5000	115	34.4
	5001–8000	34	10.2
	8000 Above	13	3.9
Education	High school and below	19	5.7
	Junior college	25	7.5
	Undergraduate	256	76.6
	Postgraduate and above	34	10.2
Occupation	full-time student	258	77.2
	Production / Sales / Marketing	11	3.3
	Administration / Human resources / Customer service	14	4.2
	Audit / Office / R & D	13	3.9
	Management / Consultant	5	1.5
	Professionals (such as accountants, lawyers, architects, medical staff, journalists, etc.)	6	1.8
	Teacher	8	2.4
	Other	19	5.7
Total		334	100

### Research variables

Based on the existing related research concepts, the dimension (variable) of the research model is defined according to the research theme, and the observation index, i.e. item, is set according to this. The operational definitions of the five structural planes are shown in Table 2.

**Table 2:** The operational definitions

Variables		Measurement item	Reference
Consumers’ Network Involvement		Consumers’ network involvement refers to the time users contact the network and their familiarity with the network.	Bellman et al., [79]
Community Expertise		Community expertise refers to the professionalism, usefulness and trust of the information provided by community members to other members.	Moorman et al., [80]
Constellation Atmosphere	Community Support	Community support means that individuals establish social networks and interact with others in the community to obtain emotional support and information help.	Bock et al., [81]
	Community Cohesion	Community cohesion is the state of the community as a social community. This state is either the common interests and mutual assistance and cooperation among members, or mutual trust and collective identity, which is helpful to coordinate the community to reach an agreed goal.	Sampson et al., [82]
	Community Contribution Recognition	Community contribution recognition means that community members share knowledge and express views, which are recognized by other members, and can meet the internal needs of members, such as pride, self-improvement and community status promotion.	Dholakia et al., [83]
Blogger Trust		Members identify with the organization and are willing to establish a long-term relationship with it.	Robinson [84]
Constellation Infatuation		Having a high degree of behavioral participation and emotional investment in the infatuated object, and obtaining a great sense of pleasure from the infatuated object.	Borup et al.; Hmoud et al., [48–49]

### Measurement

This study adapted existing research scales with appropriate adjustments for the constellation context, while meeting the requirement of structural equation modeling that each construct should have at least three items. Each construct in this study has three or more measurement items. Specifically, community support and community contribution recognition were adapted from Kim [85], community cohesion from Sampson [82], consumer internet involvement from Citrin et al. [86], community professional knowledge from Doney and Cannon [87], blogger trust from Turban [88], and constellation obsession from Williams et al. [89]. All questions in the questionnaire used a seven-point Likert scale, where 1 = strongly disagree, 2 = disagree, 3 = slightly disagree, 4 = neutral, 5 = slightly agree, 6 = agree, and 7 = strongly agree. The specific measurement scale is shown in Table 3:

**Table 3:** Variable measurement option design

Variables	Code	Measurement item	Reference
Community Support	CS1	Constellation community members are willing to help me solve problems	Kim [85]
	CS2	Constellation community members treat me sincerely	
	CS3	Constellation community members will give me valuable suggestions when I am in trouble	
	CS4	Constellation community members will encourage me when I encounter setbacks	
Community Cohesion	CC1	Members of the constellation community are willing to help each other as much as they can	Sampson [82]
	CC2	Most constellation community members have a high spirit of participation	
	CC3	The relationship between members of the constellation community is harmonious	
	CC4	I am an important part of the constellation community	
Community Contribution Recognition	CCR1	Active members of the constellation community will be more respected and loved	Kim [85]
	CCR2	Active members of the constellation community will be appropriately rewarded	
	CCR3	Active members of the constellation community are more likely to be accepted when sharing information	
	CCR4	You will be more willing to accept the suggestions of active members of the constellation community	
Consumers’ Network Involvement	CNI1	The constellation community is important to me	Citrin et al. [86]
	CNI2	The constellation community is valuable to me	
	CNI3	The constellation community is in need for me	
	CNI4	The constellation community is attractive to me	
	CNI5	He constellation community is closely related to my life	
Community Expertise	CE1	The content in the constellation community has depth	Doney and Cannon [87]
	CE2	The content in the constellation community is professional	
	CE3	The content in the constellation community is trustworthy	
	CE4	The content in the constellation community is authoritative	
Blogger Trust	BT1	I have confidence in the ability of constellation bloggers	Turban [88]
	BT2	I believe that the knowledge contributed by constellation bloggers is true and reliable	
	BT3	By browsing the articles published by constellation bloggers, I trust bloggers more	
	BT4	In general, I trust constellation bloggers	
Constellation Infatuation	CI1	When I take out my mobile phone, I will habitually click on the constellation page	Williams et al., [89]
	CI2	I feel anxious if I don’t look at the constellation for a long time	
	CI3	I think constellations often exceed my expected time	
	CI4	I’m used to knowing my situation through constellations	
	CI5	I’m used to knowing my luck through constellations	

Prior to the formal survey, two rounds of pre-tests were conducted to ensure the scientific rigor and reliability of the measurement instruments. The first round of qualitative pre-testing was conducted in November 2021, involving 50 active users from astrology communities who completed questionnaires and interviews. Three management experts and two senior astrology community managers were invited to evaluate the content validity of the items. User feedback was also collected regarding item wording, completion time, and questionnaire structure. Based on the first-round feedback, the research team optimized the wording of 12 items, removed 3 redundant items, and adjusted the questionnaire structure.

The second round of quantitative pre-testing was conducted in early December 2021 with 100 users meeting the screening criteria. Data analysis using SPSS 26.0 demonstrated good reliability and validity of all scales: In terms of reliability, Cronbach’s α coefficients for astrology obsession (α = 0.892), community atmosphere (α = 0.865), professional knowledge (α = 0.883), internet involvement (α = 0.871), and influencer trust (α = 0.894) were all above 0.85, with composite reliability (CR) values all exceeding 0.8. Regarding validity, content validity correlations from expert assessment were all above 0.8, the KMO value was 0.857 with significant Bartlett’s test of sphericity (*p* < 0.001), factor loadings were all above 0.7, AVE values exceeded 0.5, and the square root of AVE for each latent variable was greater than inter-variable correlations, indicating good content validity, construct validity, convergent validity, and discriminant validity. Item analysis showed that all items had critical ratio (CR) values above 3.0 and item-total correlations above 0.4, indicating good item discrimination. Additionally, Harman’s single-factor test showed that the first factor explained 28.6% of the variance, below the 40% threshold, suggesting common method bias was within acceptable limits.

Based on the second round pre-test results and feedback, the research team made minor adjustments to the wording of 4 items before finalizing the formal questionnaire. The results of both pre-tests demonstrated that the measurement instruments possessed good reliability and validity, making them suitable for the formal survey.

## Analysis and results

In this study, Smart-PLS 4.0 software is used for data analysis. Firstly, the reliability and effectiveness of the measurement model are evaluated, and then the structural model is tested. PLS is adopted because it is suitable for discussing the causal relationship between structural variables and can deal with model structure and project measurement at the same time. And, PLS has loose requirements for the normality and randomness of variables, and loose requirements for the normality and randomness of variables, which is suitable for dealing with variable relationships with abnormal data distribution. Meanwhile, it has the advantages of analyzing complex prediction models, reducing measurement errors and avoiding collinearity. In this study, the number of samples is 334 which meets the recommended criteria and makes it suitable for PLS analysis.

### Common method bias (CMB) assessment

This study evaluated and controlled the potential impact of Common Method Bias (CMB) through multiple approaches. First, at the research design stage, anonymous questionnaires were employed, and the survey process emphasized the authenticity of responses and the absence of right or wrong answers, reducing social desirability bias and response bias at the source [90]. Second, during the data analysis phase, Harman’s single-factor test was used to assess the possibility of CMB. The results showed that the first unrotated principal component explained X% (exceeding 50%) of the total variance. Although this result might suggest potential CMB influence, previous research has indicated that Harman’s single-factor test has low sensitivity, and relying solely on this method to judge CMB may have limitations [90–91]. To further verify the impact of CMB, this study introduced multiple control variables (demographic variables including gender, age, education level, etc.) and incorporated them into the structural equation model analysis. The analysis results showed that after controlling for these variables, the model’s path coefficients and significance levels remained almost unchanged, indicating that CMB did not substantially affect the relationships between core variables [92–93]. Finally, this study also employed the Common Latent Factor (CLF) method for supplementary CMB testing. After introducing the common latent factor into the model, changes in path coefficients did not exceed 0.2, further indicating that the impact of CMB was within an acceptable range [94]. Therefore, this study concludes that the influence of common method bias on core research findings is minimal and can be considered negligible.

### Validity and reliability assessment

This study analyzed five dimensions: astrological atmosphere, consumer internet involvement, community professional knowledge, influencer trust, and astrological obsession, examining the measurement model through both convergent and discriminant validity. Convergent validity was assessed using composite reliability and Average Variance Extracted (AVE). Composite reliability reflects the internal consistency among all measurement factors of a latent variable, while AVE is an effective statistical measure for examining the internal consistency of latent variables. Generally, a measurement model is considered to have good convergent validity when composite reliability exceeds 0.7 and AVE exceeds 0.5. Discriminant validity was examined by comparing the square root of AVE for each latent variable with its correlation coefficients with other factors. As shown in Table 4, the factor loading values for each variable ranged between 0.782 and 0.919, meeting statistical standards [95]. The AVE values of the study’s constructs ranged from 0.722 to 0.821, demonstrating good convergent validity [96].

**Table 4:** Reliability and convergent validity of constructs

	Items	Factor loading	Cronbach’s Alpha	rho_A	Composite Reliability	Average Variance Extracted (AVE)
BT	BT1 BT2 BT3 BT4	0.907 0.891 0.919 0.908	0.927	0.928	0.948	0.821
CC	CC1 CC2 CC3 CC4	0.879 0.866 0.884 0.782	0.875	0.880	0.915	0.729
CR	CR1 CR2 CR3 CR4	0.875 0.819 0.890 0.871	0.887	0.889	0.922	0.747
CS	CS1 CS2 CS3 CS4	0.895 0.923 0.903 0.900	0.926	0.927	0.948	0.819
CI	CI1 CI2 CI3 CI4	0.837 0.821 0.837 0.855	0.872	0.875	0.912	0.722
NI	NI1 NI2 NI3 NI4 NI5	0.889 0.901 0.928 0.889 0.872	0.938	0.939	0.953	0.803
PK	PK1 PK2 PK3 PK4	0.893 0.901 0.913 0.879	0.919	0.921	0.942	0.804

Note: BT = Blogger Trust; CC = Community Cohesion; CR = Community Recognition; CS = Community Support; CI = Constellation Infatuation; NI = Network Involvement; PK = Professional Knowledge

### Discriminant validity of measurement model

Discriminant validity refers to the distinguishability between different measurement variables. If the arithmetic square root of the AVE of a variable is greater than its correlation coefficient with other variables, it indicates that it has good discriminant validity (see Table 5).

**Table 5:** Discriminant validity

	BT	CA	CI	CNI	CE
BT	***0.906***				
CA	0.639	***0.804***			
CI	0.645	0.505	***0.835***		
CNI	0.704	0.733	0.672	***0.896***	
CE	0.745	0.652	0.667	0.780	***0.897***

Note: (1) The diagonal bold value is the square root of AVE of each dimension, and others are the correlation coefficients between dimensions. (2) CA = Constellation Atmosphere; CNI = Consumers’ Network Involvement; CE = Community Expertise; BT = Blogger Trust; CI = Constellation Infatuation

### Structural model analysis

In the data analysis, this study calculated the Variance Inflation Factor (VIF) values for all predictor variables. The results showed that all variables had VIF values below 5 (with the highest value being 4.962). This result meets the common assessment criteria (VIF < 5), indicating that there are no serious multicollinearity issues in the model [97].

**Fig. 2:**
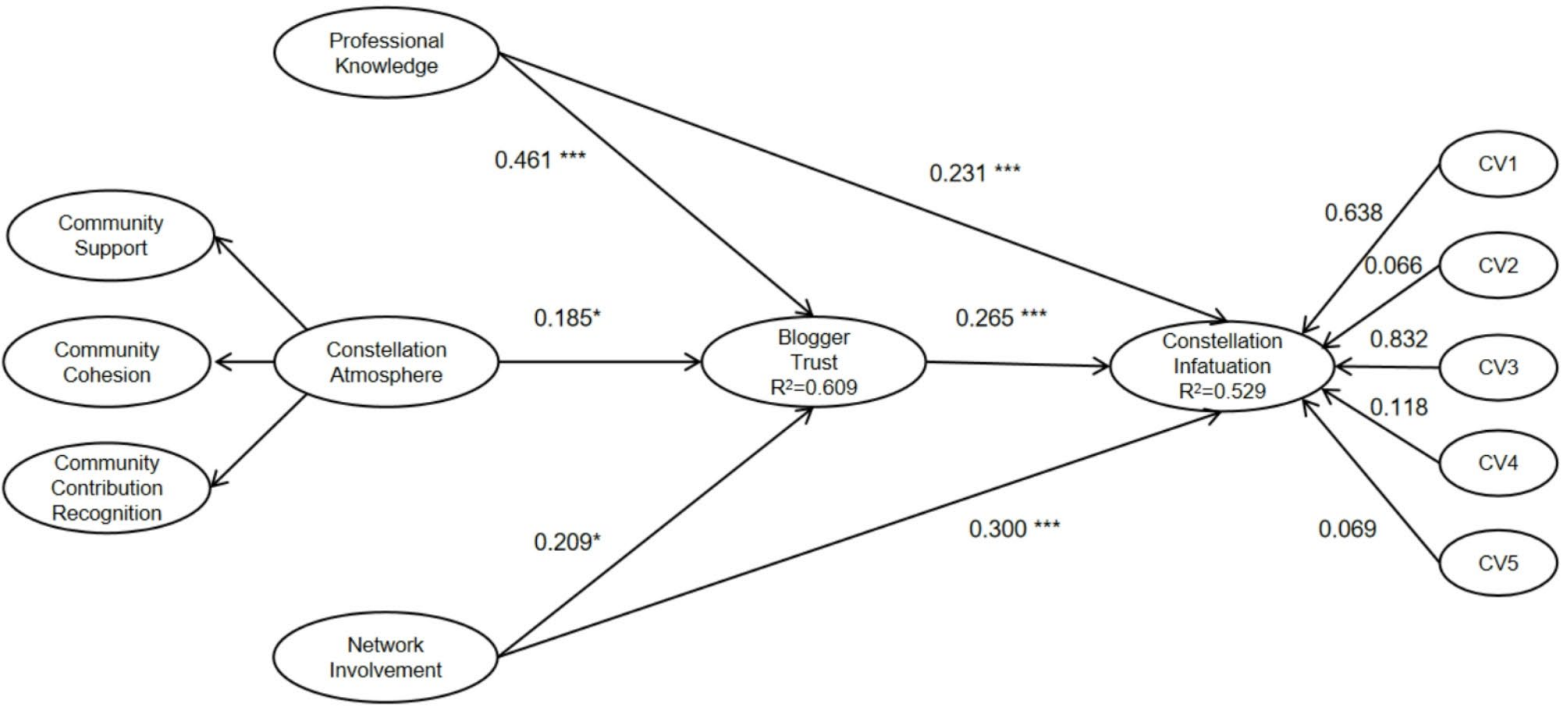
Standardized path coefficient and significance. Note: *p-value < 0.05; **p-value < 0.01; ***p-value < 0.001; CV1 = Gender; CV2 = Age; CV3 = Educational Level; CV4 = Monthly Disposable Income; CV5 = Occupation

After confirming the absence of multicollinearity issues in the model, this study proceeded with path analysis of the structural equation model. Based on the verification of scale reliability and validity, PLS statistical software was used to test the theoretical model. The path coefficients, t-values, significance levels, and hypothesis test results of the research model are shown in Table 6; Fig. 2. On the whole, the theoretical model of the research hypothesis fits well with the data, and the theoretical model is acceptable.As can be seen from Table 6; Fig. 2, the six hypotheses proposed in this study are supported. The path analysis results show significant support for all hypothesized relationships. Specifically, “Constellation Atmosphere (CA)” significantly and positively influences “Blogger Trust (BT)” (path coefficient = 0.185, T = 2.481, *P* = 0.013), supporting H1; “Professional Knowledge (PK)” significantly and positively influences “Blogger Trust” (path coefficient = 0.461, T = 6.357, *P* < 0.001), supporting H2. Additionally, “Professional Knowledge” directly and significantly influences “Constellation Infatuation (CI)” (path coefficient = 0.231, T = 3.283, *P* = 0.001), supporting H3; “Network Involvement (NI)” significantly and positively influences both “Blogger Trust” (path coefficient = 0.209, T = 2.382, *P* = 0.017) and “Constellation Infatuation” (path coefficient = 0.300, T = 3.541, *P* < 0.001), supporting H4 and H5 respectively. Finally, “Blogger Trust” significantly and positively influences “Constellation Infatuation” (path coefficient = 0.265, T = 3.599, *P* < 0.001), supporting H6.

Furthermore, the R² values in the model indicate that “Blogger Trust” is explained by 60.9% (R²=0.609) of the variance through three factors: “Constellation Atmosphere,” “Professional Knowledge,” and “Network Involvement,” while “Constellation Infatuation” is explained by 52.9% (R²=0.529) of the variance through “Professional Knowledge,” “Network Involvement,” and “Blogger Trust.” This indicates strong explanatory power of the model.

To evaluate the overall quality of the PLS-SEM model, this study employed the Goodness-of-Fit (GOF) index proposed by Tenenhaus et al. [98]. The GOF index provides a comprehensive measure of the model’s overall fit by combining the results of both the measurement model (AVE) and structural model (R²).

The GOF calculation formula is as follows:

$$GOF = \sqrt {AVE \times {R^2}} = \sqrt {0.778 \times 0.569} \approx 0.641$$

A GOF value exceeding 0.36 indicates a large effect size for model fit. Thus, the research model demonstrates strong overall goodness-of-fit, suggesting robust explanatory power for the empirical data.

**Table 6:** Model hypothesis test results

Hypothesis	Variable relationship	Standardized path coefficient	T Statistics	*P* Values	Accept/Reject
H1	CA-> BT	0.185	2.481	0.013	Accept
H2	PK-> BT	0.461	6.357	0.000	Accept
H3	PK-> CI	0.231	3.283	0.001	Accept
H4	NI-> BT	0.209	2.382	0.017	Accept
H5	NI-> CI	0.300	3.541	0.000	Accept
H6	BT-> CI	0.265	3.599	0.000	Accept

Note: 1. CA = Constellation Atmosphere; CNI = Consumers’ Network Involvement; CE = Community Expertise; BT = Blogger Trust; CI = Constellation Infatuation. 2.*p-value < 0.05; ** p-value < 0.01; *** p-value < 0.001

### Mediating effect test

In this study, the multilevel regression analysis method is used to construct a linear model step by step to test the mediating effect of blogger trust. The analysis results are shown in Table 7. According to the research results reported in Table 7, we can conclude that blogger trust (BT) is part of the mediating variables of constellation atmosphere (CA), consumers’ network involvement (CNI) and constellation infatuation (CI).

**Table 7:** Mediating effect test

Hypothesis	Variable relationship	Standardized path coefficient	VAF	T Statistics	*P* Values	Bias-corrected percentile bootstrap confidence intervals (95%)	Accept/Reject
H7	CA-> BT-> CI	0.049	35.76%	2.47	0.014	(0.010, 0.090)	Accept
H8	PK -> BT-> CI	0.122	14.21%	3.214	0.001	(0.057, 0.202)	Accept
H9	NI -> BT-> CI	0.055	32.00%	1.639	0.102	(0.007, 0.136)	Reject

Note: 1. CA = Constellation Atmosphere; CNI = Consumers’ Network Involvement; CE = Community Expertise; BT = Blogger Trust; CI = Constellation Infatuation.2.*p-value < 0.05;** p-value < 0.01;*** p-value < 0.001

In this study, we employed the Bias-corrected Percentile Bootstrap method to test the mediating effects, reporting standardized path coefficients, Variance Accounted For (VAF), T-values, P-values, and confidence intervals. The results showed that the indirect effect of “Constellation Atmosphere (CA)” on “Constellation Infatuation (CI)” through “Blogger Trust (BT)” was significant (path coefficient = 0.049, T-value = 2.47, P-value = 0.014, 95% confidence interval [0.010, 0.090]), with the mediating effect accounting for 35.76% of the total effect, supporting hypothesis H7. Similarly, the indirect effect of “Professional Knowledge (PK)” on “Constellation Infatuation (CI)” through “Blogger Trust (BT)” was also significant (path coefficient = 0.122, T-value = 3.214, P-value = 0.001, 95% confidence interval [0.057, 0.202]), explaining 14.21% of the variance, supporting hypothesis H8. However, the indirect effect of “Network Involvement (NI)” on “Constellation Infatuation (CI)” through “Blogger Trust (BT)” was not significant (path coefficient = 0.055, T-value = 1.639, P-value = 0.102, 95% confidence interval [0.007, 0.136]), thus H9 was not supported.

In summary, the research results confirmed significant mediating effects of “Blogger Trust” in the relationships between both “Constellation Atmosphere” and “Professional Knowledge” with “Constellation Infatuation.” However, “Network Involvement” did not significantly influence “Constellation Infatuation” through “Blogger Trust.” This result might be attributed to weak path coefficients or the absence of potential mediating variables in the model design. For instance, network involvement might need to influence blogger trust through intermediate variables such as “Content Attractiveness” or “Interest Matching.” Additionally, the measurement dimensions of NI might be too broad, failing to accurately reflect consumers’ specific involvement level in constellation-related content.

## Discussion

### Discussion of findings

This study explores the formation mechanism of user infatuation behavior in virtual constellation communities. The direct effects model demonstrates significant positive relationships between community atmosphere, professional knowledge, network involvement, and constellation infatuation. The indirect effects model validates the mediating role of blogger trust.

Results indicate significant positive relationships between community environmental characteristics and constellation infatuation. Specifically, professional knowledge shows the strongest impact on constellation infatuation (β = 0.461, *p* < 0.001). This finding extends Lu et al.‘s [36] perspective on professional content influencing user dependence and deepens Li et al.‘s [37] theoretical framework of professional knowledge affecting user behavior through dual cognitive and emotional pathways. Unlike previous studies such as Burtch et al. [38] that primarily focused on single dimensions of knowledge dissemination, our study reveals that professional knowledge promotes deep user engagement through the combined effects of systematic theoretical frameworks and practical interpretations.

Community atmosphere demonstrates a significant positive influence on blogger trust (β = 0.185, *p* < 0.05). This aligns with King et al.‘s [50] organizational climate theory while extending Leimeister et al.‘s [52] findings on virtual community atmosphere affecting trust building. Similar to Zhao et al.‘s [46] perspective, our study validates the mechanism of community atmosphere influencing trust formation through emotional connection, cognitive identification, and behavioral interaction dimensions.

The results confirm the crucial mediating role of blogger trust. We confirm Wang & Chan-Olmsted’s [41] proposition that trust is established through continuous interaction and value identification. Specifically: First, blogger trust plays a significant partial mediating role between community atmosphere and constellation infatuation (indirect effect = 0.049, VAF = 35.76%). This supports Fan & Lederman’s [44] dual-dimensional theoretical framework of cognitive and emotional trust. Unlike previous studies such as Zhao et al. [68] focusing on single trust dimensions, our study reveals comprehensive mediating effects of trust. Second, blogger trust also serves as a partial mediator in the relationship between professional knowledge and constellation infatuation (indirect effect = 0.122, VAF = 14.21%). This extends Ortiz et al.‘s [43] theory on bloggers as opinion leaders influencing user behavior, emphasizing trust’s key role in knowledge dissemination. Furthermore, this study responds to Srivastava et al.‘s [45] call for deeper exploration of constellation infatuation formation mechanisms. We put forward a comprehensive theoretical framework integrating community environmental characteristics (professional knowledge, community atmosphere) and user characteristics (network involvement), complementing Zhao & Ha’s [46] research on unique psychological characteristics of constellation infatuation.

Control variable analysis shows that demographic characteristics do not significantly influence constellation infatuation. This strong evidence supports Hmoud et al.‘s [49] emphasis on user behavioral characteristics’ core role in infatuation formation. Similar to Borup et al.‘s [48] findings, highly infatuated users primarily demonstrate their characteristics through sustained cognitive investment and emotional attachment.

### Practical application

This study proposes a comprehensive practical framework aimed at enhancing the operational effectiveness and user experience of virtual constellation communities. The framework provides specific and feasible implementation suggestions for virtual community operators from three dimensions: content development, community governance, and user guidance.

High-quality professional content forms the core foundation of constellation community development. Community operators should establish a systematic knowledge base of astrological theory, ensuring content professionalism and accuracy. Meanwhile, they need to develop practical, life-oriented content that provides easily understandable and applicable astrological interpretation guidance. Establishing regular content update mechanisms is also crucial, not only maintaining community vitality but also satisfying users’ continuous demand for new content. Additionally, implementing strict content review standards helps control content quality and ensures maintained professionalism.

A positive community atmosphere promotes the establishment and maintenance of user trust. First, clear community guidelines need to be established, specifying interaction rules to create a positive and rational discussion environment. Second, designing reasonable point systems and badge mechanisms encourages quality user interactions. Regular themed activities can strengthen community cohesion and promote communication and interaction among users. Meanwhile, establishing timely feedback channels and seriously addressing user opinions and suggestions helps continuously optimize community experience.

Enhancing user engagement requires systematic guidance strategies. Differentiated participation paths should be designed for users with varying levels of network involvement to meet the needs of different user groups. Bloggers should be encouraged to engage in regular in-depth communication with users, enhancing sense of belonging and trust. Establishing a complete user level system provides clear growth paths for users, stimulating motivation for continued participation. Furthermore, providing personalized content recommendations based on user interests and needs improves the precision of user experience.

Through systematic implementation of these suggestions, this framework aims to comprehensively enhance the professionalism, interactivity, and user stickiness of virtual constellation communities. It’s important to note that implementing these practical suggestions requires continuous investment and systematic planning from community operators to achieve expected results. Meanwhile, operators should maintain sensitivity to market changes, timely adjust and optimize implementation strategies to ensure the community’s sustainable and healthy development.

### Conclusions

This study constructs a formation mechanism model of user constellation infatuation behavior in virtual communities, revealing the interactive relationships among community environmental characteristics, user characteristics, and constellation infatuation. The findings demonstrate that community professional knowledge and community atmosphere are significantly positively correlated with user constellation infatuation behavior, with blogger trust playing an important mediating role. This indicates that environmental characteristics and user characteristics in virtual communities have crucial influences on the formation of constellation infatuation.

Specifically, positive community atmosphere and professional astrological knowledge can help users establish trust in bloggers, thereby promoting the formation of constellation infatuation behavior. Users’ network involvement, as an important individual characteristic, also plays a positive role in this process. These findings not only enrich the theoretical implications of virtual community user behavior research but also provide valuable guidance for community operation practices. Through establishing a theoretical model, this study systematically explains the internal mechanisms of users developing constellation infatuation in virtual communities, laying a foundation for subsequent research.

### Limitations and expectations

Some limitations of this study are: First, the cross-sectional research design limits causal inference between variables. We have to point out that the directional relationships between community environmental characteristics, user characteristics, and constellation infatuation are primarily based on theoretical deduction, lacking longitudinal empirical support. Second, it should be noted that the research sample mainly comes from constellation community users on specific social platforms. We concentrate only on convenience samples, which may introduce selection bias and may not fully represent all constellation community user groups. Third, this study was primarily conducted in the Chinese cultural context, the results may be limited in cross-cultural generalizability. Fourth, we have to point out that this study did not fully consider individual difference characteristics of users, such as cognitive styles and values, which might influence the formation of infatuation behavior.

Based on these limitations, we tentatively put forward the following suggestions for future research: First, it seems that adopting a longitudinal research design could better reveal causal relationships between variables. This may be achieved by tracking changes in user behavior over time, combining quantitative and qualitative research methods to deeply understand the formation mechanism of user infatuation behavior.

Second, future research could consider introducing richer potential variables or contextualized measurement indicators to further explore the working mechanism of user network involvement. For example, attention could be paid to specific indicators such as users’ online time distribution, interaction behavior patterns, and content production tendencies, or consider psychological characteristic variables such as users’ cognitive needs, social motives, and entertainment needs. These indicators can help measure different dimensions of user network involvement more precisely and understand its influence paths on constellation infatuation in depth. Third, we suggest expanding the sample scope to include users from different types of virtual communities to validate the universality of this research model. Moreover, conducting cross-cultural comparative studies to explore similarities and differences in user infatuation behavior across different cultural backgrounds. This is particularly important for understanding the role of cultural factors in virtual community user behavior.

Overall, despite these limitations, continued deepening and expansion through future research will help develop a more comprehensive understanding of the formation mechanism of virtual community user infatuation behavior, providing more valuable theoretical guidance for virtual community operation and management.

## Data Availability

The datasets used and analyzed during the current study are available from the corresponding author on reasonable request.

## References

[1] Kanász N. A brief history of the development of modern psychological astrology. Int J Jungian Stud. 2024;16(2):127–54. 10.1163/19409060-bja10035.

[2] Das A, Sharma M, Kashyap H, Gupta S. Fixating on the future: an overview of increased astrology use. Int J Soc Psychiatry. 2022;68(5):925–32. 10.1177/00207640221094155. 35510634 10.1177/00207640221094155

[3] Clements P. Astrology, modernity, and the project of self-identity. Cult Relig. 2020;21(3):259–79. 10.1080/14755610.2022.2093234.

[4] Lupton D, Pedersen S, Thomas G. Parenting and digital media: from the early web to contemporary digital society. Sociol Compass. 2016;10(9):730–43. 10.1111/soc4.12398.

[5] Punathambekar A, Mohan S. Social media platforms. BioScope: South Asian Screen Stud. 2021;12(2):170–3. 10.1177/09749276211026180.

[6] Nicholson B, Nielsen P, Sæbø J. Digital platforms for development. Inform Syst J. 2021;31(1):1733–55. 10.1111/isj.12364.

[7] Highfield T, Leaver T. Instagrammatics and digital methods: studying visual social media, from selfies and GIFs to memes and emoji. Communication Res Pract. 2016;2(1):47–62. 10.1080/22041451.2016.1155332.

[8] Chen J. Research on the communication strategy and realization mode of constellation culture IP. Journalism Commun. 2023;11(3):462–9. 10.12677/JC.2023.113070.

[9] Mogul D, Henderson M, Bridges J. Expanding the Facebook platform to engage and educate online communities. Am J Gastroenterol. 2017;113(4):457–8. 10.1038/ajg.2017.450. 29206814 10.1038/ajg.2017.450

[10] Siudikienė D, Jokūbauskienė S. Expression of knowledge sharing in virtual communities of interest: a reading community-based research. Inform Media. 2023;20231:1–15. 10.15388/im.2023.95.64.

[11] Fan P. The shaping and influence of virtual communities on adolescent identity in the social media era. Media Communication Res. 2023;4(7):1–12. 10.23977/mediacr.2023.040709.

[12] Verhulst B, De Wolf R, Evens T, Abeele MV. Unlock a better life: Here’s how! A critical inquiry into how life coaches gain capital and shape legitimacy using Instagram’s affordances. First Monday. 2024;29(1):1–18. 10.5210/fm.v29i1.13181.

[13] Archer A, Robb CM. Influencers as role models. Celebrity Stud. 2024;15(1):128–42. 10.1080/19392397.2024.2341594.

[14] Mehrabian A, Russell JA. An Approach to Environmental psychology. MIT Press; 1974.

[15] Lv P, Zhu X, Tian H. The impact of social sharing based on virtual community on the purchase intention of Gen Z. Oper Res Fuzziology. 2024;14(1):1–12. 10.12677/orf.2024.141009.

[16] Hossain MS, Rahman MF, Zhou X. Impact of customers’ interpersonal interactions in social commerce on customer relationship management performance. J Contemp Mark Sci. 2021;2021(1):1–12. 10.1108/JCMARS-12-2020-0050.

[17] Blight MG, Ruppel EK, Schoenbauer K. Sense of community on Twitter and Instagram: exploring the roles of motives and parasocial relationships. Cyberpsychology Behav Social Netw. 2017;20(5):314–9. 10.1089/cyber.2016.0505. 10.1089/cyber.2016.0505 28498041

[18] Zhang J, Qi S, Lyu B. A receiver perspective on knowledge sharing impact on consumer–brand relationship in virtual communities. Front Psychol. 2021;12(1):685959. 10.3389/fpsyg.2021.685959. 34707529 10.3389/fpsyg.2021.685959 PMC8542675

[19] Xu Y, Wang W. An exploration of users’ intention to continuously use English social platforms in colleges and universities based on the SOR model. Appl Math Nonlinear Sci. 2024;9(1):1–15. 10.2478/amns-2024-0249.

[20] See-To E, Rio D, P. A., Ho KKW. Social media effects in virtual brand communities. Int J Syst Service-Oriented Eng. 2016;6(2):66–88. 10.4018/IJSSOE.2016040104.

[21] Sukma N, Leelasantitham A. Factors affecting adoption of online community water user participation. Hum Behav Emerg Technol. 2022;20221:1–10. 10.1155/2022/1732944.

[22] Tabish M, Yu Z, Thomas G, Rehman SA, Tanveer M. How does consumer-to-consumer community interaction affect brand trust? Front Environ Sci. 2022;10(1):1–10. 10.3389/fenvs.2022.1002158.

[23] Luo C, Lan Y, Luo X, Li H. The effect of commitment on knowledge sharing: an empirical study of virtual communities. Technol Forecast Soc Chang. 2020;156(1):120438. 10.1016/j.techfore.2020.120438.

[24] Carvalho A, Fernandes T. Understanding customer brand engagement with virtual social communities: a comprehensive model of drivers, outcomes and moderators. J Mark Theory Pract. 2018;26(1):23–37. 10.1080/10696679.2017.1389241.

[25] Wu JJ, Chen YM, Talley PC, Kuo KM. Does online community participation contribute to medication adherence? An empirical study of patients with chronic diseases. Int J Environ Res Public Health. 2021;18(10):5100. 10.3390/ijerph18105100. 34065820 10.3390/ijerph18105100 PMC8150755

[26] Koys DJ, DeCotiis TA. Inductive measures of psychological climate. Hum Relat. 1991;44(3):265–85. 10.1177/001872679104400305.

[27] Volman M, Gilde J. The effects of using students’ funds of knowledge on educational outcomes in the social and personal domain. Learn Cult Social Interact. 2021;28(1):100472. 10.1016/j.lcsi.2021.100472.

[28] Bergstrand K, Mayer B. The community helped me: Community cohesion and environmental concerns in personal assessments of post-disaster recovery. Soc Nat Resour. 2020;33(3):1–20. 10.1080/08941920.2019.1696871. 10.1080/08941920.2019.1709002 PMC8457511 34556897

[29] Haumann T, Quaiser B, Wieseke J, Rese M. Footprints in the sands of time: a comparative analysis of the effectiveness of customer satisfaction and customer-company identification over time. J Mark. 2014;78(6):78–102. 10.1509/jm.12.0520.

[30] Javed S, Rashidin MS, Zhu M, Xu Z, Jian W, Zuo S. Combined effects of drivers and impact of customer satisfaction on brand loyalty: the contingent effect of social trust. SAGE Open. 2021;11(1):1–11. 10.1177/21582440211003566.

[31] Zyberi I, Polo A. Impact of service and e-service quality, price and image on the trust and loyalty of the electronic banking customers. Reg Sci Inq. 2021;13(1):59–68.

[32] Couper, M., Alexander, G. L., Zhang, N., Little, R. J., Maddy, N., Nowak, M. A.,…Cole Johnson, C. (2010). Engagement and retention: Measuring breadth and depth of participant use of an online intervention. Journal of Medical Internet Research, 12(4),e52. 10.2196/jmir.1430. 10.2196/jmir.1430 PMC3056524 21087922

[33] Liu Z, Song T. Big data analysis and user behavior prediction of social networks based on artificial neural network. CIT J Comput Inform Technol. 2024;32(1):1–12. 10.20532/cit.2023.1005756.

[34] Kaplan S, Fisher Y. The role of the perceived community social climate in explaining knowledge-workers staying intentions. Cities. 2021;111(1):103105. 10.1016/j.cities.2020.103105.

[35] Lahti H, Lyyra N, Hietajärvi L, Villberg J, Paakkari L. Profiles of internet use and health in adolescence: a person-oriented approach. Int J Environ Res Public Health. 2021;18(13):6972. 10.3390/ijerph18136972. 34209886 10.3390/ijerph18136972 PMC8296941

[36] Lu Y, Li Y, Wei J. Simple but logical: risk knowledge design and its impact on engagement in online knowledge communities. J Knowl Manage. 2022;27(1):31–46. 10.1108/jkm-11-2021-0838.

[37] Li Y, Gou X, Hu H, Zhang H. Exploring the impact of innovation guidance on user participation in online communities: a mixed methods investigation of cognitive and affective perspectives. Front Psychol. 2022;13(1):1011837. 10.3389/fpsyg.2022.1011837. 36248547 10.3389/fpsyg.2022.1011837 PMC9554656

[38] Burtch G, Lee D, Chen Z. The consequences of generative AI for UGC and online community engagement. SSRN Electron J. 2023;2023(1):1–42. 10.2139/ssrn.4521754.

[39] Zhong E, Fan W, Yang Q. User behavior learning and transfer in composite social networks. ACM Trans Inform Syst. 2022;34(4):1–20. 10.1145/3556143.

[40] Bagdi H, Pothabathula SV, Sharma L, Bulsara HP. The global market upsurge in web traffic and revenues during the epidemic: an exploratory research of e-learning companies. Int J Dev Issues. 2023;22(3):456–71. 10.1108/ijdi-06-2023-0147.

[41] Wang R, Chan-Olmsted S. Brand communication through social media influencers: trust building and trust transfer mechanisms. Int J Bus Communication. 2024;61(1):1–25. 10.1177/23294884241255911.

[42] Li Y. Traffic monetization on community e-commerce platforms: trust in virtual communities and its effect on actual purchase. Technol Forecast Soc Chang. 2021;169(1):120579. 10.1016/j.techfore.2021.120579.

[43] Ortiz J, Chih W, Teng H. Electronic word of mouth in the Taiwanese social networking community: participation factors. Internet Res. 2017;27(4):1058–84. 10.1108/IntR-09-2016-0276.

[44] Fan H, Lederman R. Online health communities: how do community members build the trust required to adopt information and form close relationships? Eur J Inform Syst. 2017;27(1):62–89. 10.1080/0960085X.2017.1390187.

[45] Srivastava A, Gupta T, Akhtar MS, Chakraborty T. Critical behavioral traits foster peer engagement in online communities. bioRxiv. 2023;20239:556577. 10.1101/2023.09.06.556577. 10.1371/journal.pone.0316906 PMC11729953 39804944

[46] Zhao J, Ha S. Behavioral patterns and dynamics in online niche communities. J Comput Social Sci. 2023;6(2):423–42. 10.1007/s42001-023-00192-3.

[47] Hur I, Cousins KC, Stahl B. A critical perspective of engagement in online communities. Eur J Inform Syst. 2019;28(5):523–48. 10.1080/0960085X.2019.1620477.

[48] Borup J, Graham CR, Archambault LM. Academic communities of engagement: an expansive lens for examining support structures in blended and online learning. Education Tech Research Dev. 2020;68(2):807–32. 10.1007/s11423-020-09744-x.

[49] Hmoud M, Swaity H, Daher W. High school students’ engagement in biology in the context of XR technology. IEEE Access. 2023;11(1):137053–66. 10.1109/ACCESS.2023.3338176.

[50] King KM, Rodriguez J, Louis L, Idoate R, Frankel E. Benefits of leveraging community-academic partnerships to plan and implement research institutes. J Clin Translational Sci. 2024;8(1):1–8. 10.1017/cts.2024.245.

[51] Campbell J, Shaul K, Slagle KM, Sovic D. Sustainable community development through peer-to-peer learning in the online and in-person classroom. Int J Sustain High Educ. 2024;25(2):321–38. 10.1108/ijshe-07-2023-0321.

[52] Leimeister JM, Ebner W, Krcmar H. Design, implementation, and evaluation of trust-supporting components in virtual communities. J Manage Inform Syst. 2023;21(4):101–35. 10.1080/07421222.2005.11045825.

[53] Lu Y, Zhao L, Wang B. Exploring factors affecting trust and purchase behavior in virtual communities. IEEE Symp Adv Manage Inform Globalized Enterprises. 2022;20221:1–6. 10.1109/AMIGE.2008.ECP.11.

[54] Huang HC. Enhancing doctoral learning through virtual communities of practice: an autoethnographic perspective. Front Educ. 2024;9(1):1347052. 10.3389/feduc.2024.1347052.

[55] Moy M, Feldstein A. Fostering digital communities. Networked Learn Conf. 2024;14(1):8090. 10.54337/nlc.v14i1.8090.

[56] Barnett S, Jones SC, Caton T, Iverson D, Bennett S, Robinson L. Implementing a virtual community of practice for Family Physician Training: a mixed-methods case study. J Med Internet Res. 2014;16(3):e83. 10.2196/jmir.3083. 24622292 10.2196/jmir.3083 PMC3967123

[57] Harris L, Earl G, Beale N. (2012). Building Personal Learning Networks through Event-Based Social Media: a Case Study of the SMiLE Project. Semantic Scholar. https://consensus.app/papers/building-personal-learning-networks-through-event-based-harris-earl/47c226620fa054e9868e1705608be47e

[58] Ardichvili A, Page V, Wentling T. Motivation and barriers to participation in virtual knowledge-sharing communities of practice. J Knowl Manage. 2003;7(1):64–77. 10.1108/13673270310463626.

[59] Wang EST, Chen LSL, Tsai BK. Investigating member commitment to virtual communities using an integrated perspective. Internet Res. 2012;22(2):199–210. 10.1108/10662241211214566.

[60] Vassileva J. Motivating participation in social computing applications: a user modeling perspective. User Model User-Adapt Interact. 2012;22(1–2):177–201. 10.1007/s11257-011-9109-5.

[61] Zhang H, Liu Y, Su S. Schrödinger’s cat—parallel experiences: exploring the underlying mechanisms of undergraduates’ engagement and perception in online learning. Front Psychol. 2024;15:1354641. 10.3389/fpsyg.2024.1354641. 39081380 10.3389/fpsyg.2024.1354641 PMC11287126

[62] Vătămănescu E-M, Alexandru VA, Lăzăroiu G. Capitalizing online knowledge networks: from individual knowledge acquisition towards organizational achievements. J Knowl Manage. 2022;26(4):860–79. 10.1108/jkm-04-2022-0273.

[63] Feng Y, Li X, Ma X, Zhu Z, Chen K, Gao J, Xia J, Jiang R, Lu J. Using online social networks to provide a parental health-education intervention for preventing unintentional injuries among children aged 0–3 years: a randomized controlled trial and social network analysis in Shanghai, China. Front Public Health. 2023;11:1049851. 10.3389/fpubh.2022.1049851. 10.3389/fpubh.2022.1049851 PMC9875045 36711338

[64] Chiu C-M, Hsu M-H, Wang ETG. Understanding knowledge sharing in virtual communities: an integration of social capital and social cognitive theories. Decis Support Syst. 2006;42(3):1872–88. 10.1016/j.dss.2006.04.001.

[65] Hsu M, Ju TL, Yen C, Chang C-M. Knowledge sharing behavior in virtual communities: the relationship between trust, self-efficacy, and outcome expectations. Int J Hum Comput Stud. 2007;65(2):153–69. 10.1016/j.ijhcs.2006.09.003.

[66] Lin M, Hung S-W, Chen C-J. Fostering the determinants of knowledge sharing in professional virtual communities. Comput Hum Behav. 2009;25(4):929–39. 10.1016/j.chb.2009.03.008.

[67] Cai Y, Shi W. The influence of the community climate on users’ knowledge-sharing intention: the social cognitive theory perspective. Behav Inform Technol. 2020;39(11):1187–99. 10.1080/0144929X.2020.1808704.

[68] Zhao T, Lin J, Zhang Z. The influence of multi-variation in-trust web feature behavior performance on the information dissemination mechanism in virtual community. Sustainability. 2022;14(10):6122. 10.3390/su14106122.

[69] Roseline K, Gunadi W. The effect of trust in the Soco community on repurchase intention in social e-commerce. SITEKIN: Jurnal Sains Teknologi Dan Industri. 2023;20(2). 10.24014/sitekin.v20i2.22071.

[70] Huang Y. Exploration of influencing factors on mechanism of Chinese users’ continuous trust in transactional virtual community. Asian Social Sci. 2020;16(11):80. 10.5539/ass.v16n11p80.

[71] Zhang J, Ma Y, Lyu B. Relationships between user knowledge sharing in virtual community with community loyalty and satisfaction. Psychol Res Behav Manage. 2021;14:1509–23. 10.2147/PRBM.S331132. 10.2147/PRBM.S331132 PMC8488053 34616191

[72] Kim DY, Kim HY. Trust me, trust me not: a nuanced view of influencer marketing on social media. J Bus Res. 2021;134:223–32. 10.1016/J.JBUSRES.2021.05.024.

[73] Chen Y, Liu MT, Liu Y, Chang A, Yen J. The influence of trust and relationship commitment to vloggers on viewers’ purchase intention. Asia Pac J Mark Logistics. 2021;34(2):249–67. 10.1108/APJML-08-2020-0626.

[74] Maharani S, Suprayogo D. The role of parasocial interaction as a mediator in the influence between trust and beauty influencer expertise on purchase intention (study on beauty influencer Abel Cantika). World J Adv Res Reviews. 2024;22(2):2152–62. 10.30574/wjarr.2024.22.2.1596.

[75] Ma C, Zainudin SSS, Abas WAW, Feng S, Jin D. Examining the influence of media system dependency relations on user satisfaction and continuance intention in social networking services. Stud Media Communication. 2023;11(7). 10.11114/smc.v11i7.6358.

[76] Masood A, Zhang Q, Ali M, Cappiello G, Dhir A. Linking enterprise social media use, trust and knowledge sharing: paradoxical roles of communication transparency and personal blogging. J Knowl Manage. 2023;27(4):1056–85. 10.1108/JKM-11-2021-0880.

[77] Yuan Z, Cheng X, Duan Y. Impact of media dependence: how emotional interactions between users and chat robots affect human socialization? Front Psychol. 2024;15., Article 1388860. 10.3389/fpsyg.2024.1388860. 10.3389/fpsyg.2024.1388860 PMC11362029 39220396

[78] Wang X, Ji X. Sample size estimation in clinical research: from randomized controlled trials to observational studies. Chest. 2020;158(1S). 10.1016/j.chest.2020.03.010. S12-S20. 10.1016/j.chest.2020.03.010 32658647

[79] Bellman S, Lohse GL, Johnson EJ. Predictors of online buying behavior. Commun ACM. 1999;42(12):32–8. 10.1145/322796.322834.

[80] Moorman C, Zaltman G, Deshpande R. Relationships between providers and users of market research: the dynamics of trust within and between organizations. J Mark Res. 1992;29(3):314–28. 10.2307/3172742.

[81] Bock GW, Zmud RW, Kim YG, Lee JN. Behavioral intention formation in knowledge sharing: examining the roles of extrinsic motivators, social-psychological forces, and organizational climate. MIS Q. 2005;29(1):87–111. 10.2307/25148669.

[82] Sampson RJ, Raudenbush SW, Earls F. Neighborhoods and violent crime: a multilevel study of collective efficacy. Science. 1997;277(5328):918–24. 10.1126/science.277.5328.918. 9252316 10.1126/science.277.5328.918

[83] Dholakia UM, Bagozzi RP, Pearo LK. A social influence model of consumer participation in network-and small-group-based virtual communities. Int J Res Mark. 2004;21(3):241–63. 10.1016/j.ijresmar.2003.12.004.

[84] Robinson SL. Trust and breach of the psychological contract. Adm Sci Q. 1996;41(4):574–99. 10.2307/2393868.

[85] Kim JW, Choi J, Qualls W, Han K. It takes a marketplace community to raise brand commitment: the role of online communities. J Mark Manage. 2008;24(3–4):409–31. 10.1362/026725708X306167.

[86] Citrin AV, Sprott DE, Silverman SN, Stem DE. Adoption of internet shopping: the role of consumer innovativeness. Industrial Manage Data Syst. 2000;100(7):294–300. 10.1108/02635570010304806.

[87] Doney PM, Cannon JP. An examination of the nature of trust in buyer-seller relationships. J Mark. 1997;61(2):35–51. 10.1177/002224299706100203.

[88] Turban LE. A trust model for consumer internet shopping. Int J Electron Commer. 2001;6(1):75–91. 10.1080/10864415.2001.11044227.

[89] Williams DR, Patterson ME, Roggenbuck JW, Watson AE. Beyond the commodity metaphor: examining emotional and symbolic attachment to place. Leisure Sci. 1992;14(1):29–46. 10.1080/01490409209513155.

[90] Podsakoff PM, MacKenzie SB, Lee JY, Podsakoff NP. Common method biases in behavioral research: a critical review of the literature and recommended remedies. J Appl Psychol. 2003;88(5):879–903. 10.1037/0021-9010.88.5.879. 14516251 10.1037/0021-9010.88.5.879

[91] Fuller CM, Simmering MJ, Atinc G, Atinc Y, Babin BJ. Common methods variance detection in business research. J Bus Res. 2016;69(8):3192–8. 10.1016/j.jbusres.2015.12.008.

[92] Kline RB. Principles and practice of structural equation modeling. Guilford; 2015.

[93] Richardson HA, Simmering MJ, Sturman MC. A tale of three perspectives: examining post hoc statistical techniques for detection and correction of common method variance. Organizational Res Methods. 2009;12(4):762–800. 10.1177/1094428109332834.

[94] Liang H, Saraf N, Hu Q, Xue Y. Assimilation of enterprise systems: the effect of institutional pressures and the mediating role of top management. MIS Q. 2007;31(1):59–87. 10.2307/25148781.

[95] Hair JF, Hult GTM, Ringle CM, Sarstedt M. A primer on partial least squares structural equation modeling (PLS-SEM). 2nd ed. Sage; 2017.

[96] Fornell C, Larcker DF. Evaluating structural equation models with unobservable variables and measurement error. J Mark Res. 1981;18(1):39–50. 10.1177/002224378101800104.

[97] Hair JF, Black WC, Babin BJ, Anderson RE. Multivariate Data Analysis. 8th ed. Cengage Learning; 2019.

[98] Tenenhaus M, Vinzi VE, Chatelin Y-M, Lauro C. PLS path modeling. Comput Stat Data Anal. 2004;48(1):159–205. 10.1016/j.csda.2004.03.005.

